# A Practical Treatise on Fractures and Dislocations

**Published:** 1860-05

**Authors:** 


### REVIEWS AND BIBLIOGRAPHY.

*A Practical Treatise on Fractures and Dislocations.* By FRANK HASTINGS HAMILTON, M.D., Professor of Surgery in the University of Buffalo: Surgeon to the Buffalo Hospital of the Sisters of Charity; Consulting Surgeon to the Buffalo General Hospital, and to the Buffalo City Dispensary. Illustrated with two hundred and eighty-nine Wood-Cuts. Philadelphia: Blanchard & Lea. 1860. Pp. 757.

The profession throughout the country, especially those engaged at all in surgery, or where their situation involves the necessity of taking charge of every accident requiring the aid of a physician, have anxiously awaited the appearance of Dr. Hamilton's great work on fractures and dislocations. It is truly a great work; both from the amount of labor, of which it is the result, and from its immense importance to a certain portion of the profession. In his preface, the author states the condition of English literature upon the subject of his volume, showing that here a great deficiency has long existed. "The English language does not, at this moment, contain a single complete treatise on fractures and dislocations. The two small volumes of Desault, the one of Boyer, issued near the close of the last century, and translated into English early in this, may perhaps, properly enough, have been regarded as complete treatises at the time of their publication, but they certainly cannot be so now considered. The several chapters on "*Diseases and Injuries of the Bones*," contained in the *Leçons Oracles* of Dupuytren, translated in 1846, and the *Treatise on Fractures in the vicinity of Joints, and on Certain Forms of Accidental and Congenital Dislocations*, by Robert Smith, are invaluable monographs, but neither of them claim to be anything more than a collection of occasional and miscellaneous papers. The writings of Amesbury, and of Lonsdale, relate only to fractures. Even the justly celebrated quarto of Sir Astley Cooper is no more than what its title plainly declares it to be, *A Treatise on Dislocations and on Fractures of the Joints;* but since the announcement of the present volume, a translation of Malgaigne's great and crowning work on Fractures and Dislocations has been commenced by Dr. Packard, of Philadelphia, and the first volume has been placed in the hands of the American profession. Should the remaining volume be rendered into English, the gap in our literature will be measurably filled."

The reader will see that the present volume has filled a void in a most important department of our science. Such a want has long been felt by all, and keenly felt by those whose practice in cases of fracture or dislocation has not been quite satisfactory to their patients. Those of the latter class have been mulcted, some justly, and some unjustly, without the benefit of any work in the English language as received authority on the subject. We do not intend, in making these remarks, to disparage the admirable text-books on surgery which have been issued from the American press, the works of American and English authors. It would, of course, be impossible that even a subject so important as that to which the present work is devoted, should be completely exhausted in a systematic work upon surgery; we wished to show the value and importance of a complete work on fractures and dislocations, before commenting upon the treatise before us.

It is the pride of American practitioners and surgeons to say, that in the practical application of their science, and art of medicine and surgery, they rank second to those of no other country. We can certainly claim equality, if not superiority, in this regard; and we think we will be sustained in the assertion, that in the treatment of fractures, our best surgeons stand pre-eminent. In this country, Prof. Hamilton has most identified his name with this subject, and is best qualified to write a work upon it, vindicating his claim and the claims of his countrymen to a high position in this department of surgery.

As this journal is not the medium of elaborate reviews, especially of systematic treatises, we cannot give more than a mere sketch of the plan and scope of the work before us.

Part I., more than two-thirds of the volume, is devoted to the consideration of fractures, embracing thirty-five chapters; six of which treat respectively of " general division of fractures," " general etiology of fractures," " general semieology of fractures," "repairs of broken bones," "general treatment of fractures," "delayed union and non-union of broken bones." The remaining chapters treat of special accidents; in which, while all are considered, the appropriate prominence is given to those which are most commonly met with, and most important as regards diagnosis and treatment.

The chapters on fracture of the long bones, especially those of the lower extremities, are exceedingly minute and elaborate. The author's great experience in these accidents, and the minute study which he has given to the results under different surgeons, and with varied forms of apparatus, render these the most valuable portions of the work. Already has the profession reaped much benefit from Dr. Hamilton's labors in this direction; and the elaborate reports made by him to the

American Medical Association opened an interesting and comparatively new field of investigation.

Part II. embraces twenty-six chapters, treating of all forms of dislocations. In studying this section of the work, the reader will be struck with the great improvement which has taken place in this department of surgery of late years. Dexterous surgeons are now much more successful in their manipulations; and luxations which in old times inevitably brought into play the inquisitorial pulleys or adjusters, are now frequently reduced without pain, by simple manipulation. The so-called Reid's method of reduction of dislocation of the femur by manipulation is fully discussed, showing that these dislocations had occasionally been reduced without forcible extension, even as far back as the time of Hippocrates. Prof. Hamilton prepared a very elaborate paper on the literature of reduction of the os femoris by manipulation, for the *Buffalo Medical Journal*, appearing in the numbers for February and June, 1858.

Without going into an elaborate review, it would be profitless to discuss, at length, the numerous original and important contributions of the author to the subject of fractures and dislocations. Most of these are, or should be, sufficiently familiar to the profession, as Prof. Hamilton has long been known as an indefatigable investigator into all points connected with this subject. Still, a systematic treatise is quite different from the report of a detached observation; and one well known as an able investigator might fail in presenting an entire subject in the form of an authoritative work. In the work so long looked for by the profession, they cannot be disappointed; and the author has raised for himself, and for American surgery, an enduring monument. A few more such works, and a few more such workers, and America would contribute far more than her share towards the progress of our science.

